# Monthly Summary

**Published:** 1888-08

**Authors:** 


### Monthly Summary.

Amalgam in Contact with Gold.—*By Dr. W. G. A. Bonwill, Philadelphia.*—A cause of failure in this useful, though much abused article, is that many gold fillings are allowed to remain on the grinding surfaces where proximal walls subsequently decaying have compelled the use of amalgam, and it is thus brought into direct contact with the gold. Take out the gold if you want proper anchorage, without cutting so much as to expose the pulp on the proximal service. If you do not, the alloy grows dark and unsightly, and nothing is added to its value for preservation from oxidation. Besides the tooth is much better supported by all amalgam. If a gold filling needs patching at the cervix, or on any of the margins, and gold cannot be made practicable, then do it with amalgam or pink gutta-percha. I have seen to my disgust, amalgam fillings patched with gold to set up galvanic action. Oxyphosphate is a good thing on the margins of cavities where the metal would show. It stands well in conjunction; gutta-percha does not.

It is justifiable to allow a contour gold filling to remain in an opposite cavity to be filled with amalgam, for if the latter has much gold in it the color remains quite as good as if touching a similar metal. The security, however, against shocks is to wedge well before contouring, and let them keep in contact, not opening and closing a circuit by intermittent touches.

The New-Departure idea that a leak forms a battery where it is against dentos is too trifling. The chemical action, if an acid be there, would take place simultaneously on each, and would dissolve dentos, whether permitted to touch an opposite surface of amalgam or of dentos. The acid will act as a solvent, and not galvanically; and does so just as rapidly and effectually on a smooth surface, at the union of dentos and metal, as at the point of contact. No better evidence is needed to disprove incompatibility as the cause than that when an amalgam filling is placed in close contact to the proximal surface of a perfect natural crown so that the same capillary surfaces are made as in nature, decay will as surely go on as if gold had been used. If more compatible than gold, there should have been no caries. This is also a fact when a porcelain crown is placed up tightly against sound tooth structure.—*Dental Cosmos.*

---

PREJUDICE AGAINST AMALGAM.—Dr. Francis, of New York, says: There are physicians who are prejudiced against amalgam. I have had some experience with homeopathic physicians in efforts to combat their prejudices concerning amalgam fillings and rubber plates. A lady came to me many years ago, showing much excitement, declaring that I had poisoned her daughter by putting amalgam fillings in her teeth. She stated that her child had been treated by her physician for a long time, but with no benefit whatever,and he finally decided that his failure was due to the presence of mercury in her teeth, which operated against his remedies and counteracted their effect. I requested her to bring her daughter to me. She did so, and I examined her teeth, finding four or five gutta percha stoppings and two of tin foil. No amalgam had been used, and I was almost sure of the fact previously. I was quite indignant to be thus charged with employing fillings affecting the child's health, and sent a sharp message to her physician in return. There is still much prejudice against the use of amalgam, and fears that the mercury will poison the patient or cause salivation. All this I consider absurd. My chief objection to amalgam as a filling is that it is not always reliable. Though I have seen a great many such fillings which have been in the mouth for years, and done excellent service, I have seen others which in a comparatively brief period have failed, the amalgam either receding from the cavity margins or the cavity margins decalcifying around the fillings. I suppose we must be more careful what amalgam we use and how we use it. To-day a lady requested me to fill a tooth with amalgam, and I did so. The cavity was on the posterior surface of a second bicuspid. In her teeth were a number of amalgam fillings, and all doing good service. One in a superior cuspid was perfect in color, and caused no discoloration to the tooth. So far as the chemical effect of amalgam on the system is concerned, I would not take it into consideration for a moment. Let us do our work with amalgam as thoroughly as that of gold, and we shall have less of these chemical effects.—*Dental Cosmos.*

